# Differences in how women and men in a Swedish population-based sample think about sick leave: A cross-sectional vignette study

**DOI:** 10.3233/WOR-230119

**Published:** 2024-03-08

**Authors:** Gunnel Hensing, Sara Blom, Ida Björkman, Monica Bertilsson, Johan Martinsson, Lena Wängnerud, Jesper Löve

**Affiliations:** a School of Public Health and Community Medicine, Institute of Medicine, Sahlgrenska Academy, University of Gothenburg, Gothenburg, Sweden; b Institute of Health and Care Sciences, Sahlgrenska Academy, University of Gothenburg, Gothenburg, Sweden; c Department of Political Science, University of Gothenburg, Gothenburg, Sweden

**Keywords:** Social stigma, gender, depression, social norms, attitudes, sick leave

## Abstract

**BACKGROUND:**

Gender differences in attitudes towards depression gives reason to believe that sociocultural gender norms play a role in other areas.

**OBJECTIVE:**

The aim was to test (i) if the likelihood to think that sick leave with depression symptoms is not reasonable varies between women and men, and (ii) if the likelihood to think sick leave is not reasonable varies depending on the gender of the individual with depression symptoms.

**METHODS:**

A study population of 3147 participants responded to a web-survey with a written case briefly describing a man or woman with symptoms of depression. Respondents were asked if they thought it is reasonable that the person was sick listed for two weeks. Logistic regression was used to analyse the data.

**RESULTS:**

After controlling for age, education, self-rated health, and respondent’s own experience of sickness absence the adjusted OR was 1.45 (95% CI 1.25–1.67) for men being less likely to think sick-leave was reasonable. Gender difference decreased when adjusting for negative attitudes towards depression (adjusted OR 1.24, 95% CI 1.06–1.44). No difference was found between how women and men thought about sick leave in relation to the gender of the case described in the vignette.

**CONCLUSION:**

Men were more likely to think that sick leave was not reasonable with decreased OR after adjustment for negative attitudes towards depression. Gender norms might be part of the explanation for differences but are challenging to test. This study contributes to a bourgeoning research field on gendered attitudes and sick leave, in terms of theoretical reasoning and methodological choice.

## 1 Introduction

Sickness absence with mental disorders is more common in women than in men in Sweden and Scandinavia as a whole [1], as is depression [2]. The reasons for this are multifactorial but the presence of these differences in society may contribute to “gendered” notions of sickness absence and depression [3]. Gendered notions are constructions of a phenomenon as either female or male, as feminine or masculine. Such stereotypical notions can complicate recognition, acceptance, and care-seeking for common mental disorders [4]. Möller-Leimkühler [5] argues that there is a discursive linkage between depression and femininity in how depression is described, discussed, and communicated in society in relation to gender. This linkage provides men with a strong reason to hide their depression from others which in turn can hinder or delay care-seeking. In an analysis of Swedish media articles, Bengs et al. [6] concluded that descriptions of depression, its outbreak and course were “gendered”. Depression in men was described as less emotional and with a more dramatic onset while depression in women was described as more emotional, embodied (explained by biological factors such as hormones, giving birth and menopause) and relational. “Gendered” notions also seem to be present in relation to sickness absence. In an analysis of how articles in the New York Times described and discussed absenteeism over 100 years, Patton and Johns [7] found a distinct “absence culture” for women but not for men in the sense that women’s absenteeism was described as expected given women’s multiple roles as workers, mothers and partners. They suggested that this gendered discourse on women and sickness absence can contribute to gender stereotypes and possibly also to discrimination between women and men. These studies give reason to believe that sociocultural norms linked to ‘femininity’ and ‘masculinity’ play a role in attitudes and behaviour in sick leave processes related to depression [8]. Based on the reasoning above, and given the few studies that exist, the present study tests with a written vignette design (i) if the likelihood to think that sick leave with depression symptoms is not reasonable varies with gender, and (ii) if the likelihood to think sick leave is reasonable varies with gender of the individual with depression symptoms.

### 1.1 Why expect the likelihood to think sick leave is not reasonable varies with gender?

The theoretical assumption of this study is that gendered thoughts, at least partly, may be driven by adherence to sociocultural norms for masculinity [3, 9, 10]. This assumption is supported by a recent study on perceptions of typical ‘masculine’ versus ‘feminine’ traits in Sweden which showed that masculinity, even in the Swedish context of gender equity, was linked to being *agentic* (decisive, competitive, focused) whereas femininity was linked to being *communal* (caring, relationship-oriented) [11]. A recent meta-analysis of US public opinion polls, covering more than 30,000 individuals, showed similar results and demonstrated that while some gender stereotypes changed over time –such as beliefs about individuals’ competence –beliefs about men as ambitious and courageous and women as affectionate and emotional were remarkably stable [12]. Thus, even if masculinities as social practices may vary in different social contexts, notions that tap into hegemonic masculinity seem stable over time and various contexts [3, 10]. Hegemonic masculinity can be described as an accepted type of masculinity in a certain society.

Markstedt et al. [11] showed that men and women in Sweden view themselves as having a mix of feminine and masculine characteristics. At the same time, more men than women scored high (many masculine traits) on a subjective masculinity scale and, vice versa, more women than men scored high (many feminine traits) on a subjective femininity scale. Thus, it is reasonable to believe that men in Sweden, to a higher degree than women, internalise typical masculine traits (and vice versa), or at least perform in line with these traits to act in line with accepted masculinity [9]. Being “agentic”, which represented masculinity in the Swedish study mentioned above, is in many respects the opposite to being on sick leave and thus men with depression or other common mental disorders might avoid sick leave. However, internalised norms of masculinity may also make men in general more prone to holding stricter views on others with depression symptoms. In contrast, communal norms linked to femininity may lead to more lenient attitudes among women [7]. In this study, we test the effect of respondents’ gender on attitudes towards sick leave and depression. Based on the reasoning in Markstedt et al. [11] and Möller-Leimkühler [5] we hypothesise that:

H1: The likelihood to think that sick leave with depression symptoms is not reasonable varies with gender.

### 1.2 Why expect the likelihood to think that sick leave is not reasonable varies depending on the gender of the individual with depression symptoms?

In line with gender theory, perceptions may also be gendered i.e., attitudes and thoughts may differ if a situation perceived by someone involves a man or a woman [3, 9]. Studies have found gendered perceptions in how respondent view a person’s alcohol consumption in line with traditional gender stereotypes such as weakness in women and strength in men [13, 14]. Gendered perception has also been identified in organisations, connecting men’s leadership styles with strength and being agentic and women’s with being considerate and supportive [15]. A qualitative study of health care professionals’ notions on masculinity identified how the participants discussed masculinity as elusive but distinct [16]. The elusive concept contradicted the distinct descriptions of masculine and unmasculine traits, behaviour, and qualities. These examples illustrate gender stereotypes in perceptions of others (alcohol consumers, leaders, patients) which may be applicable on differential thoughts about sickness absence in men and women.

Connell [3] suggests that gender is constructed in an ongoing interplay between social relations, practices, and locations. Thus, workplaces can be formative and normative in shaping norms and behaviour such as work participation, at least to a certain degree. In a Swedish study among GPs, reasons for sickness presenteeism were reported in line with gender-stereotypes with women reporting reasons such as “concern for colleagues” and “concern for patients” to a higher degree than men [17]. Men reported “can handle it” and “money loss” to a higher degree than women.

We assume that the more severe the depression, the less important are the gender norms for how a person with depression acts in relation to sick leave. In Sweden, it is common for workplaces to be highly gendered [18] –either dominated by men or by women –and thus men that experience a reduced work capacity may carry a “double burden” of masculinity norms as being male and embedded in settings where certain norms of masculinity may be strengthened even further compared to other settings. Classical studies on masculinity suggest that employment and being a family breadwinner are prominent aspects of a hegemonic, or accepted, masculinity [3, 10]. Thus, a man with depression symptoms may be expected to “carry on” –not be on sick leave –and fulfil work life expectations to a higher degree than a woman with the same symptoms. This could take the form of a denial of the symptoms and/or avoiding acting upon the symptoms e.g., seeking healthcare, taking sick leave. A recent Swedish study found that men were less likely to seek mental healthcare compared to women, even though they reported having experienced mental conditions requiring care [19].

There are a few earlier vignette studies on gendered perceptions of mental conditions or sickness absence. In a study of mental health literacy, Swami [20] randomised a text vignette to a sample of the general population in Britain. He found that respondents, irrespective of sex, were more likely to indicate a mental condition to the female vignette and less likely to do so for the male vignette. Furthermore, women more often than men indicated a mental condition to the male vignettes, and the likelihood of recommending care-seeking was higher among women. A Norwegian study, however, found no differences between women and men in attitudes to sick leave depending on gender of the written vignettes [21]. The study included a large variety of occupations and health conditions divided into male and female vignettes. The study did not specifically measure attitudes to sick leave with mental disorders. In conclusion, we have not found any study that specifically investigated perceptions about sickness absence in persons with depression. However, based on research from Swami et al. [20] we expect both men and women to be less willing to think that sick leave is reasonable if the person with depression symptoms is a man. Following Möller-Leimkühler’s [5] suggestion that men have strong reasons to hide their depression from others, reasonably, this may spill over in attitudes towards others with such symptoms.

This leads us to hypothesise that:

H 2: The likelihood to think sick leave is not reasonable varies depending on the gender of the vignette person with depression symptoms. 

### 1.3 Attitudes to depression

It is possible that negative attitudes to depression correlate with more negative attitudes to sickness absence with depression as well. A very stable finding, consistent across differing cultural contexts, is the association between male gender and stigmatising beliefs in depression [22–25]. For example, a recent Swedish study on persons in managerial positions found that compared to women, men had higher scores on a scale that measures managerial stigma towards employees with depression [26, 27].

In summary, few studies have studied the potential role of gendered norms in sickness absence behaviour for depression symptoms. New knowledge on how the general public think about sickness absence behaviour can contribute to future development of preventive measures to reduce the gender gap; women have twice as high sickness absence as men with mental conditions [28]. The aim of this study was to analyse differences in how women and men think of sick leave after reading a vignette that illustrates a person with depressive symptoms, and to study whether the likelihood to think sick leave is reasonable or not varied with gender of the vignette person.

The hypotheses are:

H1: The likelihood to think that sick leave a couple of weeks for depression symptoms is not reasonable varies with gender. 

H 2: The likelihood to think that sick leave a couple of weeks is not reasonable varies depending on the gender of the vignette person with depression symptoms. 

## 2 Methods

### 2.1 Study design and participants

We chose a vignette study design as an appropriate and common way of investigating perceptions and attitudes [28]. The research group developed a written case vignette based on findings from earlier qualitative studies of experiences of working while depressed [29, 30]. The case vignette described a person with common symptoms of mild to moderate depression, and the person’s work tasks (Fig. 2). The vignette was developed to be a trigger for the participants’ considerations around the question on sick leave that followed.

**Fig. 1:**
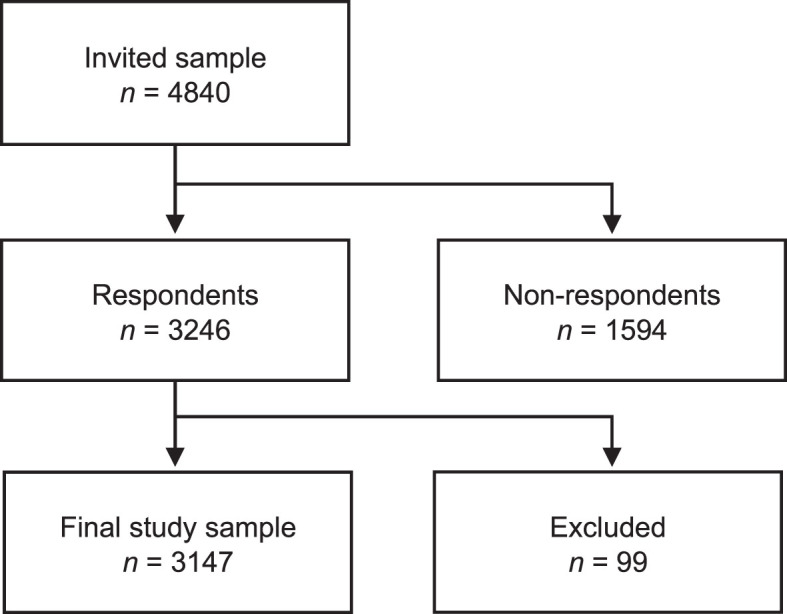
Flowchart of the final study sample.

The Laboratory of Opinion Research (LORE) at the University of Gothenburg collected the data. LORE runs online surveys approximately four times per year with the so-called Swedish Citizen Panel, a self-recruited sample from the population aged 15 years or older [31]. For this study, a sub-sample of the panel (*n* = 4840) received an e-mail invitation to an online survey. The e-mail and two reminders were sent between November 27 and December 21, 2014. The survey included questions on sociodemographic, health and political factors, and a written case vignette. The overall response rate was 67%, resulting in 3246 respondents. Non-response analysis showed that participation rates were higher among older age groups than among younger, and that survey participation rates were higher among those with university education than among other groups [31]. For this study, 62 respondents were excluded due to missing data on gender or reporting gender as “other”, 13 were excluded since they did not respond to the survey questions on sick leave, and 24 were excluded due to technical errors in the web survey. The final study sample consisted of 3147 individuals (Fig. 1).

**Fig. 2:**
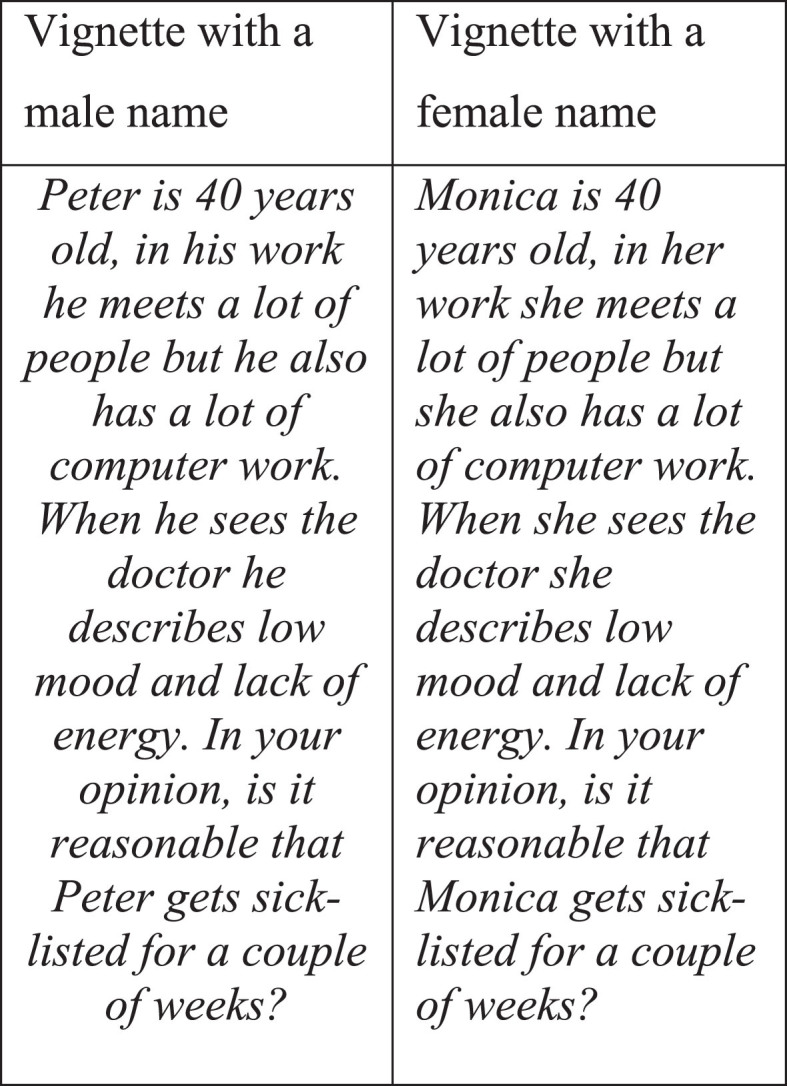
The case vignettes with a male and a female name respectively.

### 2.2 Measures

#### 2.2.1 Dependent variable

We used a random distribution of the vignette. Half of the participants received a vignette with a male first name (Peter), and the other half received a vignette with a female first name (Monica). After reading the vignette, the participants were asked “In your opinion, is it reasonable that Peter/Monica get sick-listed for a couple of weeks?” followed by four response alternatives (absolutely not, probably not, yes absolutely, yes probably). For analytical purposes, the response alternatives were dichotomised into “Not reasonable” (“no, absolutely not”, “no, probably not”) versus “Reasonable” (“yes absolutely”, “yes probably”). Since we developed the vignette for use in a multipurpose web survey, the vignette description was kept short.

#### 2.2.2 Independent variables

*Gender of the participants* was measured using self-reported data on gender with three response alternatives: woman, man, other. Those responding “other” were excluded from the study population, as the small size of the group did not allow statistical analyses (*n* = 15).

*Gender of the vignette* was measured by the gender mentioned in the vignette, as indicated by a male or female first name (Peter/Monica) followed by male/female pronouns.

#### 2.2.3 Covariates

Factors that theoretically or empirically are connected to attitudes towards sick leave and gender were chosen as covariates. Age, education, self-rated health, own sickness absence, political ideology [32–34], and negative attitudes towards depression [26] were chosen as covariates as they are connected to attitudes towards sickness absence. With the exception of age, these factors are also connected to gender, with women in Sweden having higher education, poorer self-rated health, more sickness absence, and more left-wing political ideology, and they are expected to have less negative attitudes towards depression than men [1, 18, 23–25, 35, 36].

Age was categorised into 15–30, 31–45, 46–60, and 61+ years as previously done on the same data material [34]. The level of completed education was categorised into primary or less, upper secondary, post-secondary (not university), university, and doctoral degree. Self-rated health was measured by the question “How do you rate your general health?” and categorised into good, neither good or bad, bad. Own sickness absence in the last 12 months was categorised into no sickness absence, 1–7 days, 8 days- 12 months.

Political ideology (left-right) was measured by an index with a higher score indicating more right-wing ideology (0–16 points). The scale was based on four statements responded to using a five-point Likert scale, ranging from totally agree (4) to totally disagree (0). The statements were preceded by the question “What do you think of the following propositions?”: “Sweden should receive fewer refugees”, “The tax levels should be lower”, “The taxes on CO_2_ for petrol should be raised”, and “There should be a decrease of societal income inequalities”. The coding for the third and the fourth statements were reversed in the final index.

Attitudes towards depression were measured using a subscale of the Depression Stigma Scale (DSS) [23]. The DSS is one of the most frequently used measures of stigma in population-based research on attitudes to depression [22]. The respondents were asked how strongly they agreed with different statements about depression, e.g. “Depression is a sign of personal weakness”. The nine items were followed by a five-point Likert scale ranging from strongly disagree (5) to strongly agree (1). A higher score indicated stronger personal stigma (9–45 points) which in this study was termed “negative attitudes”. Only individuals with valid data for all items received a score for the final index and were included in the relevant analyses (*n* = 3094).

### 2.3 Statistical analyses

The characteristics of the study sample were investigated by calculating frequencies and proportions of the distribution of covariates, first in the total sample, and then stratified by gender of the participant, and gender of the vignette. For continuous variables, means were calculated. Group differences in characteristics were investigated using a Chi^2^ test for proportions and an independent-samples *t*-test for continuous variables. Proportions and proportional differences with 95% Confidence Intervals (95% CI) were then calculated to test the hypotheses [37]. Two-tailed tests were applied.

The analysis was conducted for both the dichotomised outcome variables (“Not reasonable” versus “Reasonable”), and the original four categories (“No, absolutely not”, “No, probably not”, “Yes probably” and “Yes, absolutely”). Bivariate and multivariable logistic regression analyses, presented as odds ratios (ORs) with 95 % CI, were conducted controlling for covariates (reasonable = reference). Prior to the multivariable analyses, multicollinearity analyses were conducted using Spearman rho: none of the covariates had a correlation coefficient > 0.3. Covariates that had a statistically significant association (*p* < 0.05) with advising against sick leave in bivariate logistic regression analyses were included in the multivariable models. Covariates were entered in three steps: Model 1 included age and education, Model 2 added self-rated health and own sickness absence, and Model 3 added negative attitudes towards depression. Political ideology had no statistical association with the outcome and was excluded from the multivariable analyses.

In addition, bivariate logistic regression analyses were conducted to investigate whether participants who received a male vignette were more likely to think that sick leave is not reasonable than those who received a female vignette. This analysis was also stratified by gender of the participant to investigate whether this was true among both men and women. No multivariable logistic regression analyses were conducted as the distribution of vignettes with male/female name was randomised.

Lastly, sensitivity analyses were conducted to investigate whether the dichotomisation into “not reasonable” versus “reasonable” sick leave had affected the results. This was conducted using ordinal regression analysis with the original four response categories as dependent variables instead of the dichotomised variable. In addition, sensitivity analyses were conducted including only those in working age (18–64 years). Analyses were conducted using IBM SPSS Statistics, version 27.

### 2.4 Ethics approval and consent to participate

The Citizen Panel has ethical approval from the Regional Ethical Review Board in Gothenburg (No: 189-14). Informed consent was obtained from all participants included in the study. All methods were performed in accordance with guidelines and regulations of relevance for this study. Three participants were born 1999, aged 15 at the time of data collection in 2014. According to the Swedish Ethics Review Law (2003:460) and the Swedish Ethical Review Authority, persons aged 15–18 years of age do not need parents’ permission to participate in research.

The main objective of the study concerned attitudes to depression and stigma which also was given as an overall information apart from legal and ethical issues. Thus, the participants were not presented with specific information that an aim was to explore gender differences which might have biased the responses.

## 3 Results

The study sample of 3147 individuals were 15–77 years old, and 53 % were women. Table 1 shows the characteristics of the study sample, by participant gender. Women more often had university education and had had sickness absence in the past year compared to men. There were no gender differences based on age, self-rated health, or political ideology. However, men had more negative attitudes towards depression than women.

**Table 1:** Characteristics of the study sample by participant gender (N = 3147)

	Study sample		Men		Women	
	N = 3147		n = 1486		n = 1661	
	n^a^	% ^b^	n^a^	% ^b^	n^a^	% ^b^	Chi^2^ test^c^
Age, years
15–30	287	9	148	9	139	9	n.s
31–45	705	22	379	23	326	22
46–60	976	31	498	30	478	32
61–77	1168	37	629	38	539	36
Education, completed
Primary or less	167	5	97	6	70	5	*
Upper secondary	686	22	410	25	276	19
Post-secondary	393	13	230	14	163	11
University	1787	57	856	52	931	63
Doctoral degree	107	3	64	4	43	3
Health, self-rated
Good	2412	77	1276	77	1136	77	n.s
Neither good nor bad	494	16	271	16	223	15
Bad	230	7	109	7	121	8
Sickness absence last 12 months
No sickness absence	2259	72	1257	76	1002	68	*
1–7 days	583	19	284	17	299	20
8 days-12 months	288	9	113	7	175	12
	Mean		Mean		Mean		*T*-test ^c^
Negative attitudes towards depression^d^	16		17		14		*
Political ideology (left-right)^e^	7		7		7		n.s

^a^Minor inconsistencies between the frequencies and the total study sample due to missing internal data. ^b^Proportions, by column. Valid proportions, missing values excluded. Proportions were rounded to the nearest integer, therefore the total does not always sum up to 100%. ^c^Statistically significant difference (* = *p*≤0.05) based on Pearson’s Chi^2^ test for proportions, and independent-samples *t*-test for continuous variables. ^d^Higher score = More negative attitudes. Range 9–45. ^e^Higher score = More right-wing ideology. Range 0–16.

As intended, the randomised distribution of the vignette showed that 50 % (*n* = 1582) received the vignette with a male name, and 50% (*n* = 1565) with a female name. No differences in sociodemographics, health, or political characteristics was observed based on this randomisation (Appendix: Additional file 1).

Table 2 shows that a higher proportion of men thought that sick leave is not reasonable compared to women (55.7 versus 46.3 %). This proportional difference was statistically significant (9.5%, 95% CI 6.0–12.9%). However, there was no difference in the proportion of participants regarding thoughts about sick leave by gender of the vignette.

**Table 2:** Thoughts about sick leave, by participant gender, and gender of the vignette (N = 3147)

	Gender of the participant				Men versus women
	Men		Women		Proportional difference
	*n* = 1661		*n* = 1486		% (95% CI)
	*n*	% ^a^	*n*	% ^a^
Sick leave is not reasonable (vs. Sick leave is reasonable)^b^	926	55.7	688	46.3	9.5 (6.0 to 12.9)
No, absolutely not ^b^	140	8.4	96	6.5	2.0 (0.1 to 3.8)
No, probably not	786	47.3	592	39.8	7.5 (4.0 to 10.9)
Yes, probably	587	35.3	618	41.6	–6.3 (–9.6 to –2.8)
Yes, absolutely	148	8.9	180	12.1	–3.2 (–5.4 to –1.1)
	Gender of the vignette				Male versus female
	Male vignette		Female vignette		Proportional difference
	*n* = 1582		*n* = 1565		(95% CI)
Sick leave is not reasonable (vs. Sick leave is reasonable)^b^	818	51.7	796	50.9	0.8 (–2.7 to 4.3)
No, absolutely not^b^	135	8.5	101	6.5	2.1 (0.2 to 3.9)
No, probably not	683	43.2	695	44.4	–1.2 (–4.7 to 2.2)
Yes, probably	616	38.9	589	37.6	1.3 (–2.1 to 4.7)
Yes, absolutely	148	9.4	180	11.5	–2.2 (–4.3 to 0.0)

Frequencies, proportions and proportional differences with 95% confidence intervals. ^a^Proportions, by column. ^b^Based on the question “In your opinion, is it reasonable that Peter/Monica get sick-listed for a couple of weeks?”. Responses are presented dichotomised into “not resaonable” versus “reasonable”, and by the original response alternatives “No, absolutely not”, “No, probably not”, “Yes probably” and Yes, absolutely”.

Table 3 shows the OR for men compared to women, and secondly for those who received a male compared to a female vignette. Men were more likely to think that sick leave is not reasonable than women (crude OR 1.46, 95% CI 1.27–1.68). This result persisted when adjusting for sociodemographic variables (Model 1) and adding health variables (Model 2). However, when adding negative attitudes towards depression, the gender difference decreased (Model 3, OR 1.24 95% 1.06–1.44).

Those who received a male vignette did not score sick leave as not reasonable to a greater extent than those who received a female vignette (Crude OR 1.03, 95% CI 0.90–1.19). This was true for both men and women (results not shown).

**Table 3:** Likelihood of thinking sick leave is not reasonable, by gender of the participants, and gender of the vignette (N = 3147)

	Crude	Model 1	Model 2	Model 3
	OR (95% CI)	OR (95% CI)	OR (95% CI)	OR (95% CI)
Gender of the participant
Men vs. women	1.46 (1.27–1.68)	1.52 (1.31–1.75)	1.45 (1.25–1.67)	1.24 (1.06–1.44)
Gender of the vignette^a^
Male vs. female	1.03 (0.90–1.19)	–	–	–

Logistic regression analysis presented as crude and adjusted OR with 95% confidence intervals. Model 1: Adjusted for age and education. Included in analyses due to missing internal data: *n* = 3129. Model 2: + self-rated health, and sick leave in past 12 months. Included in analyses due to missing internal data *n* = 3112. Model 3: + negative attitudes towards depression. Included in analyses due to missing internal data: *n* = 3062. ^a^Presented as crude OR only due to the randomised distribution of vignettes with male/female name.

To summarise, the findings support hypothesis 1. Men had a higher likelihood of thinking that sick leave is not reasonable compared to women. This was partially explained by men’s more negative attitudes towards depression. The findings do not support hypothesis 2. The gender of the vignette person did not change how participants thought about sick leave.

### 3.1 Sensitivity analyses

The results from the sensitivity analyses were consistent with those presented above: Men were more likely to think that sick leave is not reasonable compared to women, also when using ordinal logistic regression analysis with the original four response categories instead of the dichotomised variable (Appendix, Additional file 2). This was also found for Models 1 and 2 in an analysis of only those aged 18–64 years (results not shown) while the final Model 3 showed a slightly lower precision with OR 1.18 (95% CI 0.99–1.41) compared to OR 1.24 (95% CI 1.06–1.44) in the presented results in Table 3, Model 3.

## 4 Discussion

In this cross-sectional study on a population-based sample we tested two hypotheses. We found support for H1 but not for H2. The likelihood for men to think sick leave with depression symptoms is not reasonable was higher than for women while the likelihood to think sick leave is not reasonable depending on the gender of the vignette person with depression symptoms did not differ between women and men.

The likelihood for men to think that sick leave in depression symptoms is not reasonable was partially explained by men’s more negative attitudes towards depression. The finding that men have more negative attitudes to depression is well in line with earlier studies on attitudes to depression in men [23–25]. In fact, differences between men and women seem to be the most consisting finding in research on depression attitudes [26, 38]. However, this is the first study showing that men think sick leave is not reasonable to a higher degree than women, even after controlling for negative attitudes. Comparisons to other studies are not possible due to the lack of corresponding research. Overall, few studies have focused on the potential role of gendered norms in sickness absence behaviour. This is notable since some of the differences between women and men in sickness absence remain also when comparing women and men in the same occupations and at similar workplaces [39]. Of interest for future studies is whether advising against sick leave corresponds to actual behaviour at workplaces such as questioning the need for sick leave, making fun of those who are off sick (e.g., he has been drunk again) or claiming that persons off sick are cheating the system.

We find it likely that the reason behind the differences reported here at least in part can be found in gendered sociocultural norms. This assumption is supported by the attenuated OR when attitudes to depression were adjusted for in the regression model. In this study, we could only provide theoretical reasoning on the mechanisms at work, but we build on different strands of research [5, 11, 12] that point to norms linked to femininity and masculinity that have societal effects. Of particular interest here is the study by Markstedt et al. [11] showing that even in contemporary Sweden masculinity is linked to being “agentic” whereas femininity is linked to being “communal”. The way individuals internalise, or relate to gendered norms, can partly explain why several studies show a persistent relationship between being male and holding stigmatising beliefs about depression. It could also explain the relationship between being male and advising against sick leave as found in this study. Future studies on gender, depression and sickness absence should benefit from including measures of gendered norms. The upside of such theoretical reasoning is that, even though sociocultural norms are persistent, they are, given sufficient impetus, open to change. Practical implications of such change might be lowered depression stigma in men, leading to an increased openness to seeking care [40], and then, if needed, sick leave. However, it must be acknowledged that a too generous attitude to being off sick might lead to unnecessary time off work. We must consider that sickness absence in women can mirror a societal way of resolving deficiencies in the work environment in female-dominated sectors, and the possible effect of women doing more unpaid work in families and households compared to men. And it is of course also important to acknowledge that the study results are based on differences in averages and likelihoods between women and men, and that noticeable differences within the group of women and men might be hidden.

We did not find support for H2, i.e., the gender of the vignette person was not associated with study participants’ thoughts about sick leave. This was irrespective of participants’ own gender. A review of population-based studies on gender differences in public beliefs and attitudes towards mental disorders found no clear pattern regarding the gender of the affected person [41]. Mastekaasa et al. [21] did not find that vignette gender was associated with how Norwegian managers evaluated the appropriateness of sickness absence, irrespective of diagnosis. On the other hand, a report from the Swedish National Audit Office found that women with mild to moderate depression were sick listed 30% more than men despite similar functional limitations and decreased capacity to work [42]. Also, Swami et al. [20] found that the public were more likely to indicate a mental condition for the female vignette. Thus, sickness absence diagnoses might play a role in these perceptions.

The absence of support for H2 may be due to the text vignette format. Gender identity and gender norms develop in complex interactions between individuals and society and scholars such as Connell [3] maintain that gender is created and upheld in everyday actions, communication, relations, and ongoing dynamics i.e., what Butler [9] labels *gender performativity*. Thus, a written case vignette, read in solitude, might not trigger gendered perception to the same extent as a video vignette or real-life encounters. The chosen names of the case vignettes are not ambiguous as regards being male (Peter) or female (Monica) but they might not be strongly linked to masculinity or femininity. Videos or names that are strongly associated with well-known celebrities that have profiled themselves as very masculine or feminine would perhaps have triggered gendered perceptions in a different way. Another interpretation of the lack of support for H2 could be that the text itself was gender-neutral, revealing no information on specific sectors or work tasks other than meeting people and having computer work which could represent a wide variety of work situations (the intention behind this was to give a case without too many associations, apart from the symptoms and struggles with work duties). Yet another explanation might be that the question was identified as “gendered” in itself and that participants wanted to reply in a gender-neutral way.

Future studies could make use of the vignette design by adding more variations in names in the case vignettes but also by adding gendered information e.g., the case person being a nurse or an engineer. It should be noted however that previous research in this area is, as previously discussed, inconclusive, and it is possible that the gender of the case vignette does not influence how the population think about sick leave. Due to the complexity of gender, understood as an interaction of sociocultural structure, notions and agency, qualitative studies could contribute to a deeper understanding of the role played by the gender of the affected person in participants’ reasoning around sick leave. This is supported by the results from Markstedt et al. [11] (see also references therein) who showed that men and women in Sweden view themselves as having a mix of feminine and masculine characteristics. This makes it reasonable to expect even more complex patterns of thoughts on sick leave if cases of ‘hyper-masculine’ men and ‘hyper-feminine’ women were separated from men and women less strongly conforming to gender typical ideals. Indeed, there are different kinds of masculinities and femininities, and in certain subgroups of men, for example among those who are younger or those with higher educational levels, other masculinity ideals than a hegemonic agentic ideal might prevail. However, before concluding that case gender does not have an association with thoughts on sick leave, new studies are needed that have a variation in how vignette cases conform to gender ideals to different degrees.

### 4.1 Methodological considerations

There are several strengths of this cross-sectional vignette study. Firstly, the written vignette was developed based on results from earlier interview studies performed with persons who had experienced depression but also with healthcare professionals. This was intended to strengthen the relevance and validity of the described symptoms and related difficulties at work. Secondly, the study was based on a population-based sample with a variation of age groups and with representation of women and men from different social strata. Thirdly, the study included several relevant covariates, making it possible to account for potential bias. Finally, it has been observed that studies in the field of gender, beliefs and attitudes on mental disorder often lack a solid theoretical foundation [22]. In this study, we used gender theories to develop the hypotheses and to discuss the findings.

The most important limitation of this study is the self-recruitment of the study sample. A random sample of the general population would have been preferable. This sample has, in comparison with a random general population sample, a higher proportion of older participants and of participants with high education, which restricts the generalisability of the findings. The high education level might also have led to an underestimation of the differences between women and men. Highly educated men have lower levels of negative attitudes towards depression compared to low-educated men [25]. It can be assumed that low-educated men would have thought sick leave is not reasonable to a greater extent than found in this study. The vignette used in this study has been discussed above regarding gender, and a possible too neutral presentation. It might also be that the presentation of depressive symptoms was too general or too mild. With mild symptoms, it might be considered better to stay at work and adjust work tasks to reduce demands. Not all respondents may have considered the symptoms as related to depression so the thoughts about sick leave could concern other types of sick leave as well.

## 5 Conclusion

In this study, men compared to women were more likely to think that sick leave for symptoms of depression is not reasonable. The difference remained but attenuated after adjustment for negative attitudes towards depression. Sociocultural norms around femininity and masculinity might be part of the explanation for the differences but are challenging to test. The findings of this study contribute to a bourgeoning research field on gendered attitudes and sick leave, in terms of theoretical reasoning and methodological choice.

## Supplementary Material

Supplementary Material

## Data Availability

The datasets used and analysed during the current study are available from the corresponding author upon reasonable request.
