# Supplementary material for: Differences in how women and men in a Swedish population-based sample think about sick leave: A cross-sectional vignette study
Source: Work. 2024 Mar 8;77(3):827–38. doi: 10.3233/WOR-230119 (PMC10977418; doi:10.3233/WOR-230119)
Supplement: Supplementary Material [file wor-77-wor230119-s001.docx]

| **Supplementary table 1. Characteristics of the study sample by the randomised distribution of male/female vignettes (N=3147)** | | | | | |
| --- | --- | --- | --- | --- | --- |
|  | Participants who received a male vignette | | Participants who received a female vignette | |  |
|  | n=1582 | | n=1565 | |  |
|  | n^a^ | %^b^ | n^a^ | %^b^ | Chi^2^ test^c^ |
| Gender, participant |  |  |  |  |  |
| Men | 843 | 53 | 818 | 52 | n.s |
| Women | 739 | 47 | 747 | 48 |  |
| Age, years |  |  |  |  |  |
| 15-30 | 135 | 9 | 152 | 10 | n.s |
| 31-45 | 339 | 22 | 366 | 23 |  |
| 46-60 | 509 | 32 | 467 | 30 |  |
| 61-77 | 591 | 38 | 577 | 37 |  |
| Education, completed |  |  |  |  |  |
| Primary or less | 78 | 5 | 89 | 6 | n.s |
| Upper secondary | 364 | 23 | 322 | 21 |  |
| Post-secondary | 202 | 13 | 191 | 12 |  |
| University | 884 | 56 | 903 | 58 |  |
| Doctoral degree | 50 | 3 | 57 | 4 |  |
| Health, self-rated |  |  |  |  |  |
| Good | 1205 | 76 | 1207 | 77 | n.s |
| Neither good or bad | 265 | 17 | 229 | 15 |  |
| Bad | 106 | 7 | 124 | 8 |  |
| Sickness absence last 12 months |  |  |  |  |  |
| No sickness absence | 1138 | 72 | 1121 | 72 | n.s |
| 1-7 days | 307 | 19 | 276 | 18 |  |
| 8 days-12 months | 132 | 8 | 156 | 10 |  |
|  | Mean |  | Mean |  | T-test ^c^ |
| Negative attitudes towards depression^d^ | 16 |  | 16 |  | n.s |
| Political ideology (left-right)^e^ | 7 |  | 7 |  | n.s |

^a^ Minor inconsistencies between the frequencies and the total study sample due to missing internal data.

^b^ Proportions, by column. Valid proportions, missing values excluded. Proportions were rounded to nearest integer, therefore the total does not always sum up to 100%.

^c^ Statistically significant difference (*= p< 0.05) based on Pearson’s Chi^2^ test for proportions, and independent-samples t-test for continuous variables.

^d^ Higher score= More negative attitudes. Range 9-45.

^e^ Higher score= More right-wing ideology. Range 0-16.

| **Supplementary table 2. Sensitivity analyses using ordinal logistic regression**^a^. **Likelihood of thinking sick leave is not reasonable, by gender of the participants, and gender of the vignette (N=3147).** | | | | |
| --- | --- | --- | --- | --- |
|  | Crude | Model 1 | Model 2 | Model 3 |
|  | OR (95% CI) | OR (95% CI) | OR (95% CI) | OR (95% CI) |
| **Gender of the participant** |  |  |  |  |
| Men vs. women | 1.44 (1.26–1.64) | 1.49 (1.31–1.71) | 1.41 (1.23–1.61) | 1.17 (1.02–1.34) |
| **Gender of the vignette^b^** |  |  |  |  |
| Male vs. female | 1.10 (0.97–1.26) | - | - | - |

Presented as crude and adjusted OR with 95% confidence intervals

^a^ Based on the question “In your opinion, is it reasonable that Peter/Monica get sick-listed for a couple of weeks?” with the response alternatives “No, absolutely not”, “No, probably not”, “Yes probably” and Yes, absolutely”.

^b^ Presented as crude OR only due to the randomised distribution of vignettes with male/female name.

Model 1: Adjusted for age and education. Included in analyses due to missing internal data: n=3129

Model 2: + self-rated health, and sick leave past 12 months. Included in analyses due to missing internal data: n=3112

Model 3: + negative attitudes towards depression. Included in analyses due to missing internal data: n=3062
